# Mycobacteria Clumping Increase Their Capacity to Damage Macrophages

**DOI:** 10.3389/fmicb.2016.01562

**Published:** 2016-10-04

**Authors:** Cecilia Brambilla, Marta Llorens-Fons, Esther Julián, Estela Noguera-Ortega, Cristina Tomàs-Martínez, Miriam Pérez-Trujillo, Thomas F. Byrd, Fernando Alcaide, Marina Luquin

**Affiliations:** ^1^Departament de Genètica i de Microbiologia, Facultat de Biociències, Universitat Autònoma de BarcelonaBellaterra, Spain; ^2^Servei de Ressonància Magnètica Nuclear and Departament de Química, Universitat Autònoma de BarcelonaBellaterra, Spain; ^3^Division of Infection Diseases, Depatment of Medicine, The University of New Mexico School of Medicine, AlbuquerqueNM, USA; ^4^Servei de Microbiologia, Hospital Universitari de Bellvitge-Institut d’Investigació Biomèdica de Bellvitge, Universitat de BarcelonaBarcelona, Spain

**Keywords:** *Mycobacterium abscessus*, virulence factors, infection of macrophages, clumps, cords, rough morphotypes, smooth morphotypes

## Abstract

The rough morphotypes of non-tuberculous mycobacteria have been associated with the most severe illnesses in humans. This idea is consistent with the fact that *Mycobacterium tuberculosis* presents a stable rough morphotype. Unlike smooth morphotypes, the bacilli of rough morphotypes grow close together, leaving no spaces among them and forming large aggregates (clumps). Currently, the initial interaction of macrophages with clumps remains unclear. Thus, we infected J774 macrophages with bacterial suspensions of rough morphotypes of *M. abscessus* containing clumps and suspensions of smooth morphotypes, primarily containing isolated bacilli. Using confocal laser scanning microscopy and electron microscopy, we observed clumps of at least five rough-morphotype bacilli inside the phagocytic vesicles of macrophages at 3 h post-infection. These clumps grew within the phagocytic vesicles, killing 100% of the macrophages at 72 h post-infection, whereas the proliferation of macrophages infected with smooth morphotypes remained unaltered at 96 h post-infection. Thus, macrophages phagocytose large clumps, exceeding the bactericidal capacities of these cells. Furthermore, proinflammatory cytokines and granuloma-like structures were only produced by macrophages infected with rough morphotypes. Thus, the present study provides a foundation for further studies that consider mycobacterial clumps as virulence factors.

## Introduction

The genus *Mycobacterium* contains important human pathogens, such as *Mycobacterium tuberculosis, M. leprae, and M. ulcerans.* Furthermore, non-tuberculous mycobacterial species, as inhabitants of the environment, are important re-emerging opportunistic pathogens (Primm et al., 2004; Falkinham, 2009, 2013; van der Werf et al., 2014). In solid media, mycobacterial strains display different colony morphotypes. Rough colony morphotypes (R) are characterized by an irregular dry surface with many wrinkles and crests, whereas smooth colony morphotypes (S) show an even, bright and moist texture (Belisle and Brennan, 1989; Muñoz et al., 1998; Rüger et al., 2014). *M. tuberculosis* have a highly conserved R morphotype, whereas non-tuberculous mycobacterial species show both morphotypes, and spontaneous S to R and R to S morphology shifts have also been described (Byrd and Lyons, 1999; Howard et al., 2006; Agustí et al., 2008; Park et al., 2015). The S to R variation is accompanied by the loss of surface glycolipids, such as glycopeptidolipids (GPL) or lipooligosaccharides (LOS) (Belisle and Brennan, 1989; Muñoz et al., 1998; Pang et al., 2013). Genetic evidence supporting the relationship between colonial morphotypes and the loss of surface glycolipids has been reported (Deshayes et al., 2008; Pawlik et al., 2013; Boritsch et al., 2016). Another phenotypic difference between S and R morphotypes is that R morphotypes show increased cellular aggregation. The bacilli of R morphotypes remain attached during replication, forming compact colonies containing structures that resemble cords (Howard et al., 2006; Agustí et al., 2008). In liquid media, R morphotypes aggregate to form clumps. Large clumps acquire cord morphologies (Julián et al., 2010; Sánchez-Chardi et al., 2011; Brambilla et al., 2012). In previous studies, the R morphotypes of *M. avium*, *M. kansasii*, *M. marinum*, and *M. abscessus* are more virulent than the S morphotypes (Belisle and Brennan, 1989; Kansal et al., 1998; Catherinot et al., 2007). The species *M. canettii*, also referred to as “smooth tubercle bacilli,” is closely related to *M. tuberculosis* and exhibits an S morphotype. In contrast to *M. tuberculosis*, isolates of *M. canettii* are rare and restricted to some regions of Africa. A recent study reported the increased virulence of a spontaneous R morphotype of *M. canettii versus* the wild S morphotype (Boritsch et al., 2016).

The identification of factors that confer more virulence to R morphotypes would increase the current understanding of the mechanisms involved in the pathogenesis of tuberculosis and other mycobacterial diseases and contribute to the development of new drugs.

A majority of the studies performed to determine the mechanisms that confer more virulence to R morphotypes have primarily focused on *M. abscessus*, a re-emerging pathogenic species that causes serious chronic pulmonary infections in patients with underlying respiratory diseases, such as bronchiectasis or cystic fibrosis (Petrini, 2006; Medjahed and Singh, 2010; Lee et al., 2015). *M. abscessus* strains isolated from humans display colonies with S and R morphotypes, and several studies have demonstrated that R morphotypes are more virulent than S morphotypes (Byrd and Lyons, 1999; Sanguinetti et al., 2001; Howard et al., 2006; Catherinot et al., 2007, 2009; Rüger et al., 2014). In *M. abscessus*, the more virulent R morphotypes have been associated with hyper-proinflammatory responses to mycobacterial Toll like receptor-2 (TLR-2) ligands expressed on the cell surface (Gilleron et al., 2008; Rhoades et al., 2009; Roux et al., 2011).

In addition to the molecules expressed on the surface of R morphotypes, the formation of clumps (only present in R morphotypes) could also play a role in the increased virulence of these morphotypes. Currently, there are no studies investigating the interactions of clumps with the host, as most infection studies are conducted using homogeneous bacterial suspensions containing isolated bacilli. To obtain non-aggregating cultures, mycobacteria are cultured in media containing detergents, such as Tween, or are subjected to physical disaggregation procedures to obtain a homogeneous suspension of isolated bacilli (Byrd and Lyons, 1999; Stokes et al., 2004; Howard et al., 2006; Catherinot et al., 2007; Bernut et al., 2014; Leisching et al., 2016). However, inside the host, mycobacteria exist in a detergent-free environment, and R mycobacteria replicates initially forming small clumps that eventually become large clumps with cording morphology. Clumps and cords are not laboratory artifacts, as the presence of the clumps and cords of *M. abscessus* and *M. marinum* have been reported in zebrafish (Clay et al., 2008; Bernut et al., 2014). Furthermore, the presence of *M. bovis* BCG cords in the cytoplasm of macrophages and the dendritic cells of mouse splenic granulomas (Ufimtseva, 2015) and clumps of *M. abscessus* in the sputum of patients with cystic fibrosis have been reported (Qvist et al., 2013). Thus, it is reasonable to assume that macrophages interact with clumps of R morphotypes and not with the isolated bacilli of R morphotypes inside the host. To verify this hypothesis, we infected macrophages with mycobacterial clumps.

Thus, the objective of the present study was to describe the initial interaction between the macrophages and bacterial clumps of *M. abscessus* R compared with that of the isolated bacilli of *M. abscessus* S. For infection studies, J774, a murine monocyte/macrophage cell line that enables the comparative study of virulence among mycobacterial strains in a homogeneous population, was used. (Ramakrishnan and Falkow, 1994; Gao et al., 2003; Indrigo et al., 2003; Tan et al., 2006; Julián et al., 2010).

The progression of infection inside macrophages was examined using confocal laser scanning microscopy (CLSM) and transmission electron microscopy (TEM). The different capacities of R and S morphotypes to kill macrophages and induce the formation of granulomas, and the secretion of IL-6 and TNF-α were determined (Welsh et al., 2008; Philips and Ernst, 2012). Previous studies have reported that pulmonary surfactant proteins A and D induce the agglutination of *M. tuberculosis* bacilli (Ferguson et al., 2006). In the present study, the interaction of clumps with dipalmitoyl phosphatidylcholine (DPPC), one of the most abundant lipids of pulmonary surfactant, was also investigated (Chimote and Banerjee, 2005).

## Materials and Methods

### Bacterial Strains

The bacterial strains used in the present study were *M. abscessus* type strain DSMZ 44196^T^, *M. abscessus* 390, *M. abscessus* BE37R, and *M. abscessus* BE48R (**Table 1**). The original *M. abscessus* type strain (DSMZ 44196^T^) displayed S colonies on agar medium (called 44196S), but after a few passages on agar medium, natural R colony mutants appeared (44196R). *M. abscessus* 390 was isolated in pure culture from an ileal granuloma in a patient with Crohn’s disease (Byrd and Lyons, 1999). *M. abscessus* 390 displayed R colonies on agar medium (390R), but after serial passages on agar medium, an S morphotype was isolated (390S) (Byrd and Lyons, 1999). *M. abscessus* BE37R and BE48R were isolated from pulmonary disease patients hospitalized at Bellvitge University Hospital in Barcelona, Spain. BE37R and BE48R were identified using polymerase chain reaction and reverse hybridization (GenoType Mycobacterium CM^®^, Hain Lifescience, Germany) and the partial sequencing of 16S rRNA. All strains were grown on trypticase soy agar (TSA; Scharlau, Spain) medium for 2 weeks at 37°C.

**Table 1:** Strains of *M. abscessus* used in the present study.

*M. abscessus* strains	Colonial morphology	Cord formation	Isolated
390S	Smooth	No	Natural mutant of 390R
390R	Rough	Yes	Isolated from an ileal granuloma in patient with Crohn’s disease
DSMZ 44196^T^ S	Smooth	No	Type strain
DSMZ 44196^T^ R	Rough	Yes	Natural mutant of DSMZ 44196^T^S
BE37R	Rough	Yes	Isolated from sputum in patient with severe pulmonary disease
BE48R	Rough	Yes	Isolated from sputum in patient with severe pulmonary disease


### Colony Morphology

To determine the ultrastructure of the colonies, the bacteria were grown on TSA for 2 weeks at 37°C. The colonies were processed for scanning electronic microscopy (SEM) as previously described (Julián et al., 2010). Briefly, the colonies were recovered from the agar plate and fixed in 2.5% (vol/vol) glutaraldehyde in 0.1 M phosphate buffer (pH 7.4) for 2 h at 4°C. Subsequently, the samples were washed four times for 10 min each in 0.1 M phosphate buffer, post-fixed in 1% (wt/vol) osmium tetroxide and 0.7% ferrocyanide in phosphate buffer, followed by washing with water, dehydration in an ascending ethanol series (50, 70, 80, 90, and 95% for 10 min each and twice with 100% ethanol), and critical-point drying with CO_2_. The samples were coated with gold and observed using an SEM EVO (Zeiss, Germany) at 15 kV.

### Aggregate Size Analysis of the Bacterial Suspensions

Colonies of *M. abscessus*, cultivated for 2 weeks, were scraped from TSA plates and vigorously shaken in a tub with glass beads (Schlesinger et al., 1990; Julián et al., 2010). The bacteria were subsequently resuspended in phosphate-buffered saline (PBS), and large aggregates were allowed to sit for 10 min. The supernatant was recovered and adjusted to the McFarland standard one suspension, followed by centrifugation at 1500 g for 10 min at 4°C. The supernatant was removed, and the pellet was resuspended in Dulbecco’s modified Eagle’s medium (Gibco, USA) supplemented with L-glutamine, high glucose, and 10% heat-inactivated fetal bovine serum (Hyclone, USA); this was considered complete medium (CM). To break up the aggregates, the suspension was sonicated in an ultrasonic cell water bath three times for 30 s each (Stokes et al., 2004; Julián et al., 2010). In a series of preliminary experiments, cell viability of each mycobacterial strain was tested after sonication in order to verify the cell counts obtained in McFarland adjustments. In all experiments, representative bacterial suspensions were serially diluted in PBS, and colony-forming units (CFU) were counted after plating on TSA.

The bacterial suspension was labeled with Phenolic Auramine (Mycobacteria Fluorescent Stain – Fluka, USA), according to the manufacturer’s instructions, and the images were generated using a TCS-SP5 CLSM (Leica, Germany) with a Plan Apo 63× (numerical aperture [NA], 1.4) oil objective, operating at a zoom of 1.8. This procedure facilitated the quantification of the bacteria number per ml in bacterial solution adjusted to the McFarland standard 1. To analyze the clump sizes, horizontal (x–z) optical sections of twenty fields for each sample were captured, and the fluorescence intensity and area were measured. To quantify the size ranges based on the number of bacilli in each clump, the area sizes were classified as described: areas ≤3 μm^2^ were considered as 1–2 bacilli, areas between 3 and 6 μm^2^ were considered as 3–4 bacilli, and areas >6 μm^2^ were considered as five or more bacilli. The images were processed using ImageJ software (National Institutes of Health, USA).

### Cell Culture

The murine macrophage cell line J774A.1 (DSMZ ACC 170) was maintained at 37°C in a 5% CO_2_-humidified atmosphere in CM containing 100 U/ml penicillin G (Laboratorios Ern, Spain) and 100 μg/ml streptomycin (Laboratorios Rech-Jofre, Spain).

### Phagocytosis Assay

For phagocytosis analyses, macrophage cells (5 × 10^5^/well) were cultured on plates (Mat Tek, USA) with CM for 24 h at 37°C in a humidified atmosphere with 5% CO_2_. The macrophages were infected with R and S morphotypes at a MOI of 10:1 and 100:1, respectively. In a set of previous experiments, we observed a more inefficient phagocytosis of the S morphotype as has been previously observed by other authors (Etienne et al., 2002; Villeneuve et al., 2003; Kocíncová et al., 2009); thus, to obtain a similar average of macrophage infection for both morphotypes, a higher MOI was used in infection experiments with the S morphotype. These MOI levels yielded between 50 and 60% of macrophages infected at 3 h.p.i. In addition, three wells per dish were left uninfected as a negative control. After infection, the CM was removed, and the macrophages were washed three times with fresh CM to extract extracellular bacteria, followed by fixation with 4% paraformaldehyde (Sigma–Aldrich, USA) in PBS for 10 min. Subsequently, the macrophages were washed with PBS and air dried for 30 min. For CLSM observation, the mycobacteria were stained with Phenolic Auramine (Mycobacteria Fluorescent Stain – Fluka, USA) according to the manufacturer’s instructions. The macrophages were labeled with 1 μl red fluorescent CellMask (Molecular Probes, USA)/ml of PBS for 10 min at room temperature and subsequently washed with PBS. The images were obtained using a TCS-SP5 CLSM (Leica, Germany) equipped with a Plan Apo 63× (numerical aperture [NA], 3.0) oil objective in horizontal (x–z) optical sections. The images were subsequently analyzed using Imaris v. 6.1.0. scientific software (Bitplane, Switzerland). Ten fields per sample (approximately two hundred macrophages) were considered. The assay was performed twice.

### Transmission Electron Microscopy (TEM)

For TEM analysis, infected macrophages were fixed with a solution containing 2% (wt/vol) paraformaldehyde (Merck, Ireland) and 2.5% (vol/vol) glutaraldehyde (Merck, Ireland) in 0.1 M phosphate buffer (Sigma–Aldrich, USA) at pH 7.4 for 1 h. After fixation, the infected macrophages were recovered using a cell scraper and were processed following conventional procedures (Lee et al., 2011). The sections were observed using a Jeol 1400 transmission electron microscope (Jeol, Japan).

### Macrophage Viability

Macrophages viability was measured by two different assays: trypan blue exclusion and CLSM assay. The macrophages were seeded onto 48-well tissue culture plates (Thermo Fisher Scientific, Denmark) at 6 × 10^4^ cells per well in CM and were incubated at 37°C in a 5% CO_2_-humidified atmosphere. After 24 h, the macrophages were infected as described above. At 3 h.p.i., the medium was removed, and the macrophages were washed three times with CM to remove extracellular bacteria and were then incubated with fresh CM at 37°C. At 24-h intervals, the infected macrophages were washed again three times to prevent bacterial overgrowth in the extracellular medium. At different time points after infection (3, 24, 48, 72, and 96 h), the supernatant was removed, and the macrophages were gently washed with PBS, trypsinized (trypsin-EDTA, PAA, Austria) for 10 min and subsequently stained with trypan blue (Gibco, USA). Viable macrophages were counted using a Neubauer chamber. Each essay was performed in triplicate, and the experiment was performed three times.

For the CLSM assay, 5 × 10^5^ macrophages per well were plated in culture dishes (Mat Tech, USA) and infected as described above. At different time points after infection (3, 24, 48, 72, and 96 h), the macrophages were labeled with 0,5 μl/ml of green fluorescent 4 mM Calcein acetoxymethyl and 0,2 μl/ml of red fluorescent 2 mM Ethidium homodimer-1, LIVE/DEAD^®^ Viability/Cytotoxicity Kit (Molecular Probes, USA) for 10 min at room temperature, and then washed with CM before being observed. Labeled macrophages were examined under a CLSM (FV1000-Olympus) using a Plan Apo 20× (numerical aperture [NA], 1.5). For the processing of the images ImageJ software (National Institutes of Health, USA) was used. Ten random fields were taken for each treatment and the mean of live cells per field was calculated.

### Granuloma-Like Structure Analysis

The macrophages were seeded onto 8-well sterile glass chamber slides (Thermo Fisher Scientific, Denmark) and infected as described above. At different time points after infection (3, 24, and 48 h), the supernatants were removed, and the samples were heat-fixed and stained using the Ziehl-Neelsen (ZN) method. Observations and images were obtained using an optical microscope (Leica, Germany) equipped with a DM500 digital camera system. For SEM analysis, glass coverslips (Nahita, Spain) were inserted in the 24-well culture plates, and the macrophages (1.2 × 10^5^/well) were subsequently seeded and infected as described above. Coverslip – adhered macrophages were processed for SEM at different time points after infection (3, 24, and 48 h) as described above.

### Cytokine Analysis

For cytokine analysis macrophages were infected with the same procedure as for viability assay. Supernatants were collected at different time points, without previous washing steps, and were centrifuged to eliminate the bacteria and/or macrophages detached from the well, in order to avoid any interference in the ELISA assays. TNF-α (R&D Systems, USA) and IL-6 (BD Biosciences, Belgium) levels were measured at different time points after infection using commercially available enzyme-linked immunosorbent assays (ELISA) according to the manufacturer’s instructions. All samples were analyzed in duplicate.

### Assay of Bacterial Suspension Exposed to DPPC

A variation of the methods of Schwab et al. (Schwab et al., 2009) was used. Briefly, in a 24-well culture plate 100 μl of 13.5 mg/ml DPPC (Sigma–Aldrich, USA) in PBS was added to each well, and 100 μl of PBS per well was added to the controls. The plate was incubated at 37°C for 30 min, and subsequently 200 μl of a bacterial suspension of *M. abscessus* 390R, prepared as previously described, was added to each well. After incubating the plate for 30 min at 37°C, the content of each well was mixed once with a micropipette and counted using CLSM as previously described.

### Statistical Analysis

Comparisons between two parameters were evaluated by Student’s *t*-test. Statistical analyses were performed using PAST3 software.

## Results

### Only R Colonies Contained Attached Bacilli Forming a Cording Structure

**FIGURE 1:**
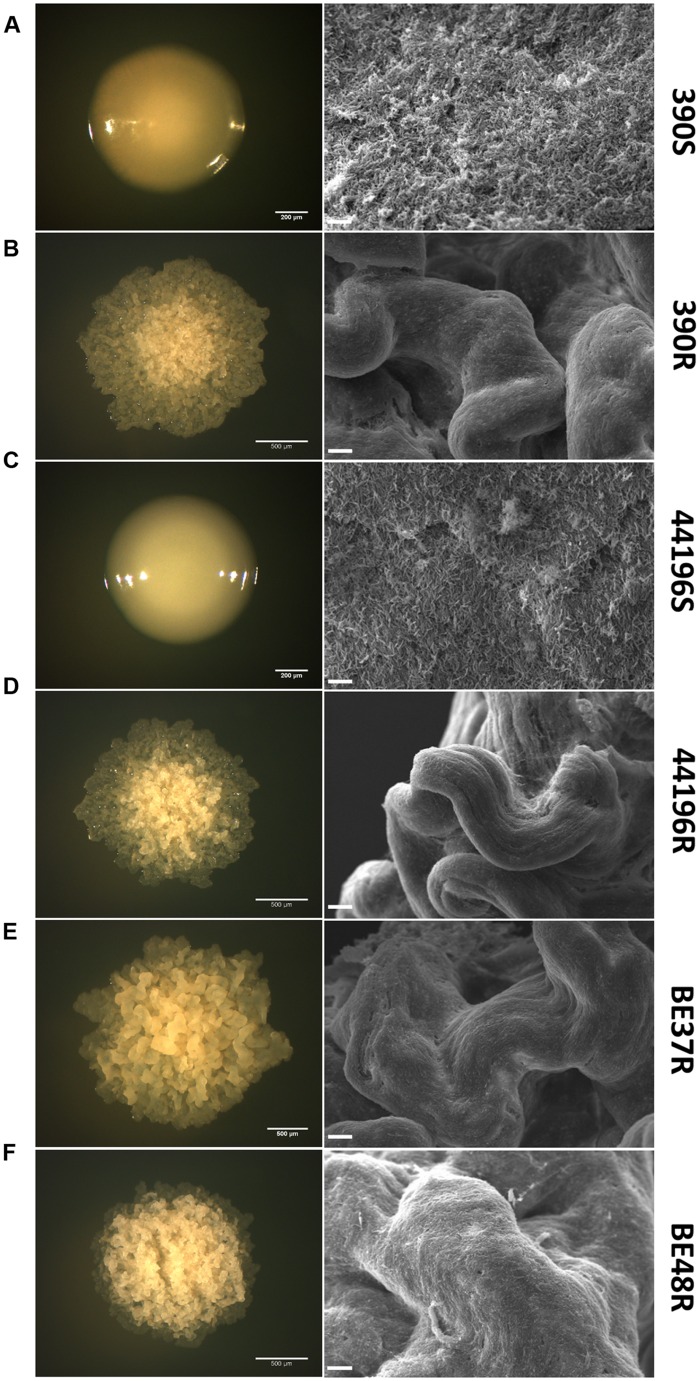
**Differences in the ultrastructure of R and S colonies.** The first column represents colonies of *M. abscessus* 390S **(A)**, 390R **(B)**, 44196S **(C)**, 44196R **(D)**, BE37R **(E)**, and BE48R **(F)** grown on trypticase soy agar plates. Bar size, 200 μm **(A,C)** and 500 μm **(B, D–F)**. The second column comprises images of colonies obtained by SEM. Bar size, 10 μm.

It was easy to differentiate R and S colonies in agar media. The R colonies were characterized by an irregular dry surface with many wrinkles and crests (**Figures 1B,D–F**), whereas the S colonies (S) showed an even, bright and moist texture (**Figures 1A,C**). SEM was used to characterize the organization of the bacilli inside the colonies. SEM of S colonies revealed masses of cells without orientation, and empty spaces were clearly visible among the single cells (**Figures 1A,C**). However, the bacilli in the R colonies were closely arranged end-to-end and side-to-side forming a cord (**Figures 1B,D–F**).

### Macrophages Infected with R Morphotypes Phagocytosed Large Clumps of Bacilli

**FIGURE 2:**
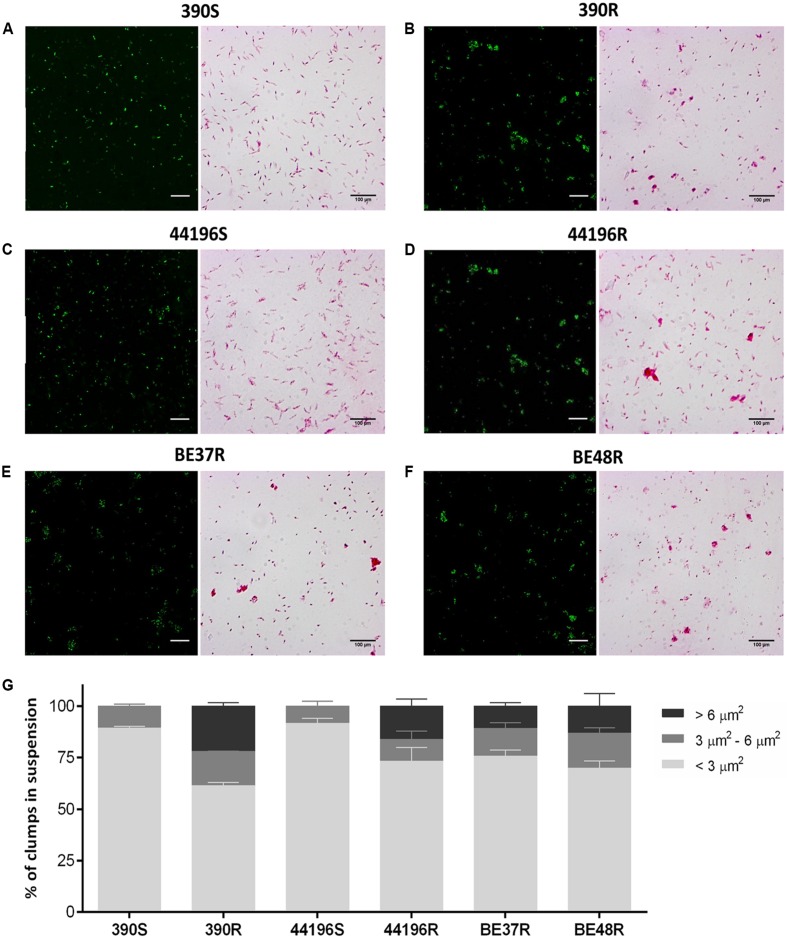
**Analysis of the aggregates in the bacterial suspensions.** **(A–F)** Images of CLSM and Ziehl–Neelsen staining showing bacterial aggregates or isolated bacilli from bacterial suspension. Bar size, 100 μm in the Ziehl–Neelsen staining images. For CLSM, the bacteria were stained with phenolic auramine (green). Bar size, 15 μm. **(A)** 390S, **(B)** 390R, **(C)** 44196S, **(D)** 44196R, **(E)** BE37R, **(F)** BE48R. **(G)** Show the results of the analysis of the percentages of clumps of different sizes in the bacterial suspensions. The results represent the mean ± SD of triplicate preparations.

The clumps in bacterial suspensions used to infect macrophages were observed using ZN staining and quantified by CLSM (**Figures 2A–F**). After disaggregating the clumps of bacilli as described above, the suspensions of S morphotypes (390S and 44196S) contained 89.4% ± 0.8 (mean ± SD) and 91.7% ± 2.3 isolated bacilli, respectively. Small aggregates of 3–4 bacilli represented 10.6% ± 0.9 of the 390S suspensions and 8.3% ± 2.3 of the 44196S suspensions (**Figure 2G**). The largest difference in the R morphotypes suspensions was the presence of aggregates of more than five bacilli that were not observed in the suspensions of smooth bacilli (**Figure 2G**).

**FIGURE 3:**
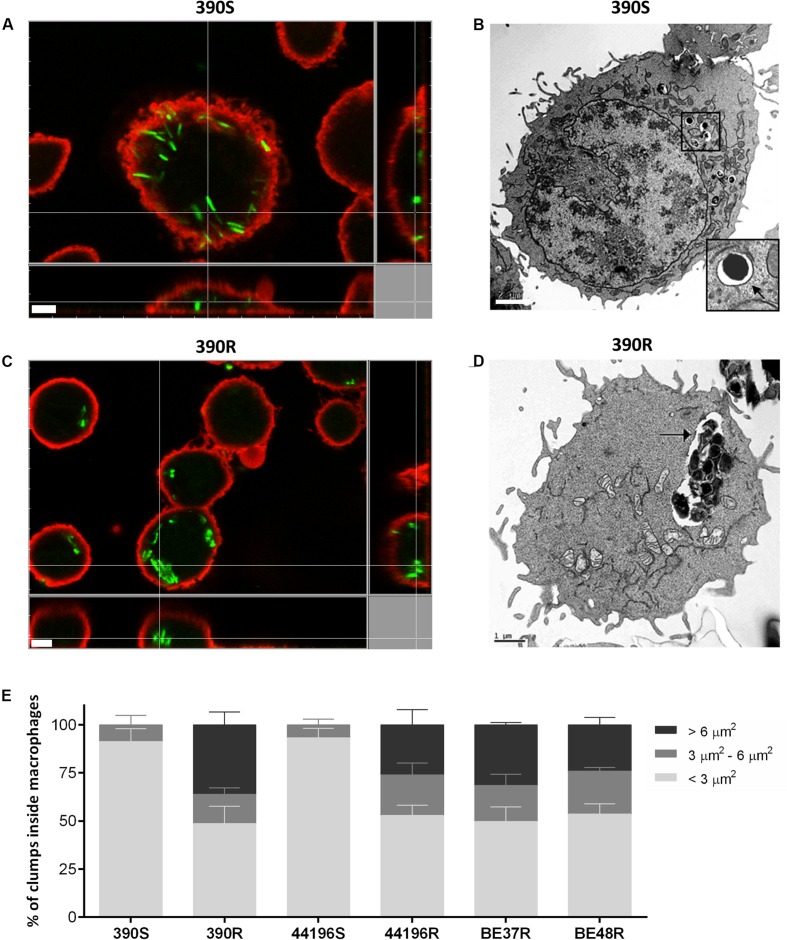
**Localization of intracellular bacteria using CLSM and transmission electron microscopy (TEM).** **(A,B)** Images of macrophages infected with *M. abscessus* 390S. In **(B)**, the membrane of phagocytic vesicle is indicated with a black arrow. Bar size, 2 μm. **(C,D)** Images of macrophages infected with 390R. **(D)** Intracellular clump of 390R inside a large phagocytic vesicle. The phagocytic vesicle membrane is indicated with a black arrow. Bar size, 1 μm. Bacteria in **(A–C)** were stained with phenolic auramine (green) and macrophage cell membranes stained with CellMask (red). Bar size, 5 μm. Similar images were obtained for the remaining strains. **(E)** Show the results of the analysis of the percentages of clumps of different sizes inside the macrophages. The results represent the mean ± SD of triplicate preparations.

The proportion of macrophages infected at 3 h.p.i. was similar for the two morphotypes (between 50 and 60%). Macrophages infected with smooth morphotypes contained mainly isolated bacilli at 3 h.p.i. (**Figure 3A**). TEM images clearly showed individual bacilli inside the phagocytic vesicles of macrophages infected with S morphotypes (**Figure 3B**). In contrast, between 24.1 and 36.1% of the bacilli observed at 3 h.p.i. inside macrophages infected with R morphotypes were organized in clumps of more than five bacilli (**Figures 3C,E**). TEM images revealed large clumps inside the phagocytic vesicles (**Figure 3D**).

### Macrophages were Rapidly Damaged by Rough Morphotypes but were Unaffected by Smooth Morphotypes

**FIGURE 4:**
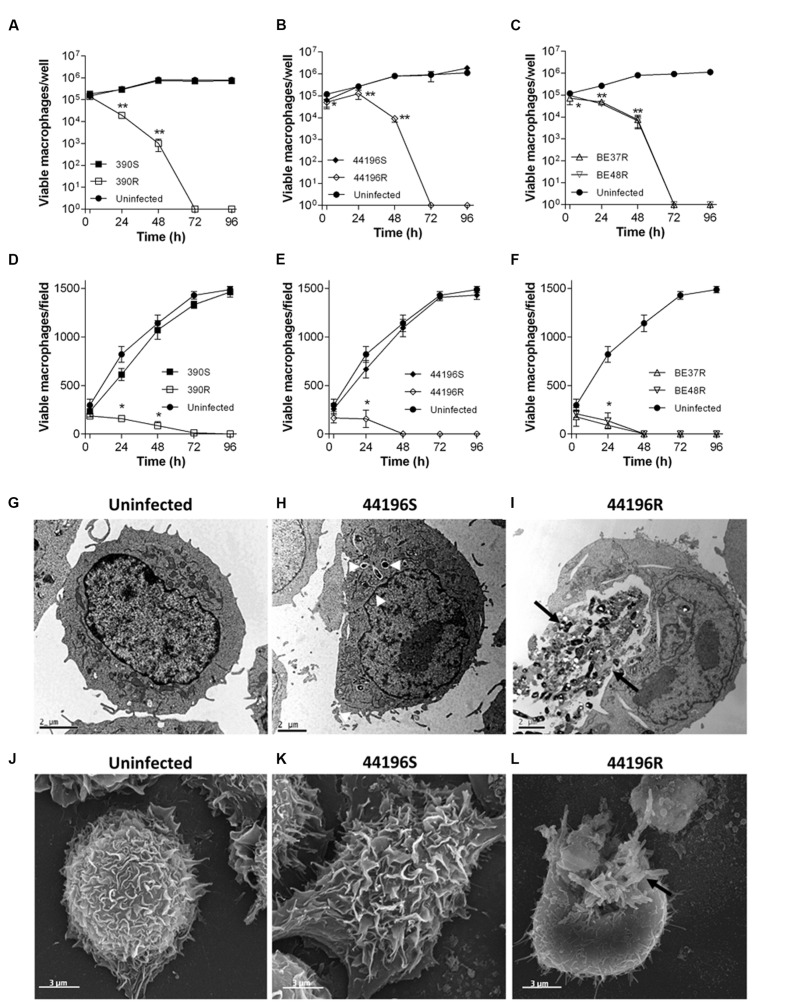
**Survival of macrophages, TEM and SEM images of uninfected and infected macrophages.** **(A–F)** Viability of macrophages infected with *M. abscessus* strains, measured using a trypan blue exclusion assay **(A–C)** and a CLSM assay **(D–F)** at different time points after infection. The data are presented as the means ± SD for triplicate wells of infected and uninfected macrophage cultures. The data are representative of one out of three independent experiments. ^∗^*P* < 0.05; ^∗∗^*P* < 0.001, significant difference compared to uninfected macrophages (Student’s *t*-test). **(G)** Representative TEM images of uninfected macrophages. **(H)** Macrophages infected with 44196S at 24 h.p.i. **(I)** Macrophages infected with 44196R at 24 h.p.i. Bar size, 2 μm. **(J)** Representative SEM images of uninfected macrophages. **(K)** Macrophages infected with 44196S at 24 h.p.i. **(L)** Macrophages infected with 44196R at 24 h.p.i. Bar size, 3 μm. Similar images were obtained for the other the strains. White arrows indicate isolated bacilli, and black arrows indicate bacterial clumps.

Similar results were obtained using the two techniques to measure the macrophages viability. R morphotypes killed 100% of infected macrophages at 48–72 h.p.i.. Both techniques gave us complementary information: while trypan blue assay provides accurate quantification of viable cells being a more sensitive test, CLSM permits rapidly compare the variation of the number of viable cells between the different morphotypes infections. However, the growth of macrophages infected with S morphotypes was unaltered after 96 h.p.i., proliferating in a similar manner to uninfected macrophages (**Figures 4A–F** and **Supplementary Figure 1**). Consistent with the viability results, macrophages infected with S morphotypes showed similar appearance to uninfected macrophages by electron microscopy at 24 h.p.i. (**Figures 4G,H,J,K**). In contrast, damaged macrophages with extruding *M. abscessus* clumps were observed in the cultures infected with R morphotypes at 24 h.p.i. (**Figures 4I,L**).

### The Death of Macrophages was Preceded by the Formation of Granuloma-Like Structures and the Production of TNF-α and IL-6

**FIGURE 5:**
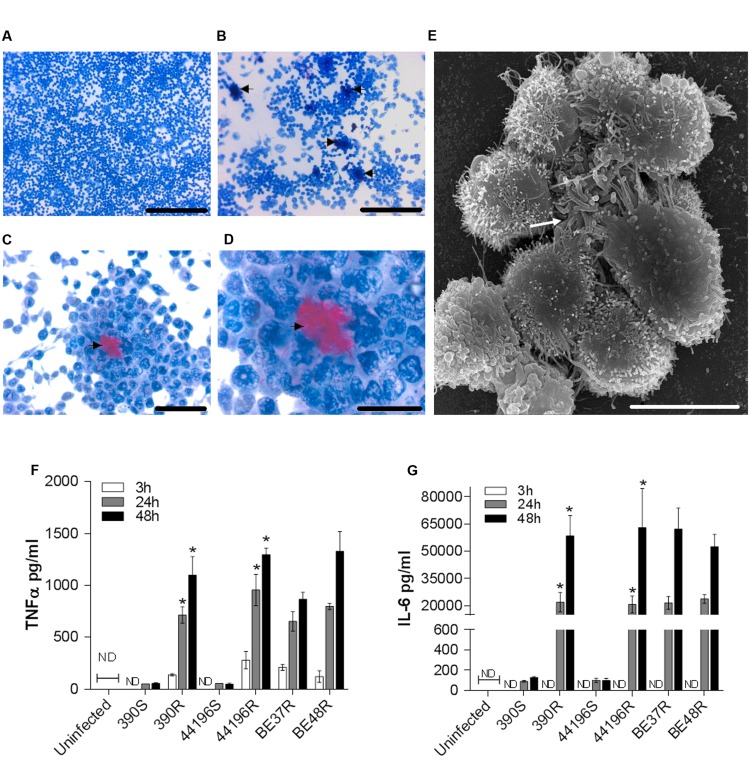
**Granuloma-like structures formed by macrophages infected with R morphotypes at 24 h.p.i. and secretion of cytokines by infected murine macrophages.** **(A–D)** Representative light microscopy images of Ziehl–Neelsen-stained macrophages. **(A)** Macrophages infected with 390S. Bar size, 250 μm. **(B–D)** Macrophages infected with 390R. The black arrow indicates clumps of bacilli stained in red. Bar sizes of 250 μm in **(B)**, 50 μm in **(C)** and 25 μm in **(D)**. **(E)** Representative SEM image of a granuloma-like structure produced around a clump of 390R. Bar size, 10 μm. White arrow indicates the bacterial clump. Similar images were obtained for the other strains. **(F–G)** The kinetics of TNFα **(F)** and IL-6 **(G)** production were analyzed using ELISA. The results represent the mean ± SD of triplicate preparations. The data are representative of one out of three independent experiments. ^∗^*P* < 0.001, significant difference between cording and its respective non-cording morfotype (Student’s *t*-test). ND, not detected.

Cultures stained with ZN at 24 h.p.i. showed no special organization of macrophages infected with smooth morphotypes (**Figure 5A**). In contrast, granuloma-like structures formed by a cluster of macrophages surrounding clumps of bacilli were observed in cultures infected with R morphotype (**Figures 5B–D**). SEM revealed the granuloma-like structures and the associated mycobacteria in greater detail (**Figure 5E**).

As granuloma formation and maintenance has been associated with the release of TNF-α and IL-6 in *M. tuberculosis*, we assessed the presence of these cytokines in the supernatants of infected macrophages. We observed that macrophages infected with R morphotypes released larger amounts of TNF-α and IL-6 at 24 and 48 h.p.i., whereas only trace amounts of these cytokines were detected in the culture supernatants of macrophages infected with S morphotypes (**Figures 5F,G**).

### The Contact with the Major Component of Pulmonary Surfactant does not Affect the Clump Size

**FIGURE 6:**
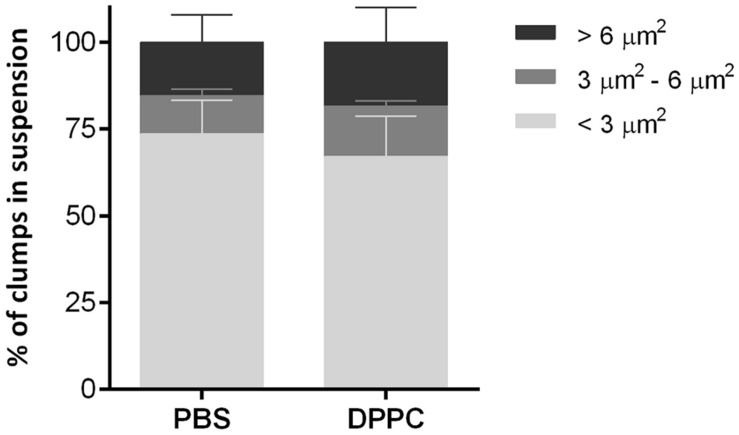
**Results of the analysis of the percentages of clumps of different sizes present in bacteria suspensions treated with dipalmitoyl phosphatidylcholine (DPPC) and the negative control with phosphate saline buffer (PBS).** The results represent the mean ± SD of triplicate preparations.

To determine whether DPPC, one of the most abundant lipids of pulmonary surfactant, could disaggregate mycobacterial clumps, a suspension of *M. abscessus* 390R was incubated with DPPC, and the formation of aggregates was comparatively quantified from the 390R suspension in PBS. No significant differences were observed between the two samples. The percentage of isolated bacilli was 67.1% ± 11.5 (mean ± SD) for the suspension treated with DPPC and 73.6% ± 9.6 for the control with PBS. Clumps with 3–4 bacilli constituted 14.3% ± 1.5 of the DPPC suspension and 11% ± 1.8 of the PBS suspension, and large aggregates constituted 18.5% ± 10 and 15.4% ± 7.8, respectively, of the clumps observed (**Figure 6**). These results correspond to the mean ± SD of the three independently performed experiments.

## Discussion

The results of the present study provide the first evidence that macrophages can phagocytose *M. abscessus* clumps, demonstrating that macrophages can interact with both clumps and isolated bacilli. No other studies have shown this capacity, as disaggregation techniques are typically used to obtain isolated R bacilli in the suspensions used to infect macrophages, or the information concerning the presence of clumps in these suspensions was omitted (Byrd and Lyons, 1999; Stokes et al., 2004; Howard et al., 2006; Catherinot et al., 2007; Bernut et al., 2014; Leisching et al., 2016). Using CLSM and TEM, we observed the presence of clumps of R morphotypes at an early stage of infection (3 h.p.i.), suggesting that the bacilli result from engulfment but not from replication inside the phagocytic vesicles. Thus, the engulfment of clumps of R morphotypes corresponds with the first R *M. abscessus*-macrophage interaction, as R bacilli form aggregates, and these aggregates have been observed *in vivo* (Clay et al., 2008; Qvist et al., 2013; Bernut et al., 2014; Ufimtseva, 2015). TEM revealed that as the clumps grow inside the phagocytic vesicles, these bacteria kill macrophages, which subsequently liberate large clumps of bacilli into the extracellular space. Previous studies have reported that these clumps grow in the extracellular space, producing large cords that cannot be phagocytosed (Bernut et al., 2014). The TEM images obtained in the present study are consistent with the 100% macrophage mortality observed for macrophages infected with R morphotypes 390R, 44196R, BE37R, and BE48R. In contrast, no effects in macrophages containing isolated S bacilli inside phagocytic vesicles were observed. In addition to the destruction of macrophages, the formation of granulomas and the release of significant amounts of proinflammatory cytokines were only observed in R morphotypes. These results are in agreement with the hypervirulence reported for 390R and 44196R in cellular cultures and animal models, in contrast with the inability of isogenic S variants (390S and 44196S) to produce illness (Byrd and Lyons, 1999; Howard et al., 2006; Catherinot et al., 2007; Bernut et al., 2014). These results are also consistent with the virulence of the respiratory isolates BE37R and BE48R. The factors that confer additional virulence to R morphotypes remain obscure, but it has been postulated that glycopeptidolipids mask immunostimulatory cell wall components, TLR-2 ligands such as phosphatidyl-myo-inositol mannosides, lipomannan and lipoarabinomannan, that enable S variants to colonize the respiratory tract of patients with cystic fibrosis or bronchiectasis (Qvist et al., 2013), prior to the switch to R morphotypes lacking glycopeptidolipids and expressing these TLR-2 ligands on the cell surface (Gilleron et al., 2008; Rhoades et al., 2009). Notably, a more recent study proposed that the lipoproteins on the cell surface of R morphotypes are responsible for TLR-2 activation and discarded the role of phosphatidyl-myo-inositol mannosides, lipomannan and lipoarabinomannan as TLR-2 activators because these factors were not detected on the cell surface of R morphotypes (Roux et al., 2011).

Thus, different groups are working on the surface exposed virulence factors that make more virulent the R morphotypes, however, most of these virulence factors are unknown yet. But the fact that the macrophage may engulf groups over five bacilli or more in a single phagocytic vesicle means that macrophage has to deal with more of these virulence factors. Our main contribution, to the knowledge of virulence mechanisms, has been to show that the macrophage can engulf such large aggregates of bacteria and therefore should be face to a major amount of virulence factors whatever they are.

Consequently, it is reasonable to assume that the accumulation of these virulence factors could overwhelm the bactericidal capabilities of these cells. The present study is a preliminary study providing a foundation for further investigations of the role of clumps in the pathogenicity of mycobacterial R morphotypes. The compounds that agglutinate mycobacterial bacilli and facilitate clump formation remain unknown because, to date, these factors have been underestimated as a result of the generalized use of homogeneous bacterial suspensions in experimental procedures.

Future studies will involve the examination of these components to identify the clumping factors as important therapeutic targets.

## Author Contributions

Conceived and designed the experiments: CB, ML-F, EJ, and ML. Performed the experiments: CB, ML-F, EN-O, CT-M, and MP-T. Analyzed the data CB, ML-F, EN-O, EJ, MP-T, and ML. Contributed reagents/materials/analysis tools TB and FA. Contributed to the writing of the manuscript: CB, ML-F, EJ, MP-T, TB, FA, and ML.

## Conflict of Interest Statement

The authors declare that the research was conducted in the absence of any commercial or financial relationships that could be construed as a potential conflict of interest.

## Abbreviations

CLSM: confocal laser scanning microscopy
CM: complete medium, Dulbecco’s modified Eagle’s medium with fetal bovine serum
DPPC: dipalmitoyl phosphatidylcholine
h. p. i.: hours post-infection
IL-6: interleukin 6
MOI: multiplicity of infection
PBS: phosphatase saline buffer
R: rough
S: smooth
SEM: scanning electron microscopy
TEM: transmission electronic microscopy
TLR-2: Toll like receptor-2
TNF-α: tumor necrosis factor α
ZN: Ziehl-Neelsen
