# Supplementary material for: Mycobacteria Clumping Increase Their Capacity to Damage Macrophages
Source: Front Microbiol. 2016 Oct 4;7:1562. doi: 10.3389/fmicb.2016.01562 (PMC5047892; doi:10.3389/fmicb.2016.01562)
Supplement: Supplementary file 1 [file Data_Sheet_1.DOCX]

Supplementary Material

**Mycobacteria clumping increase their capacity to damage macrophages**

**Cecilia Brambilla, Marta Llorens-Fons, Esther Julián, Estela Noguera-Ortega, Cristina Tomàs-Martínez, Miriam Pérez-Trujillo, Thomas F. Byrd, Fernando Alcaide, and Marina Luquin***

*** Correspondence:** Marina Luquin [(mail to: marina.luquin@uab.cat)](mailto:(mail%20to:%20marina.luquin@uab.cat))

# Supplementary Figures and Tables

## Supplementary Figures


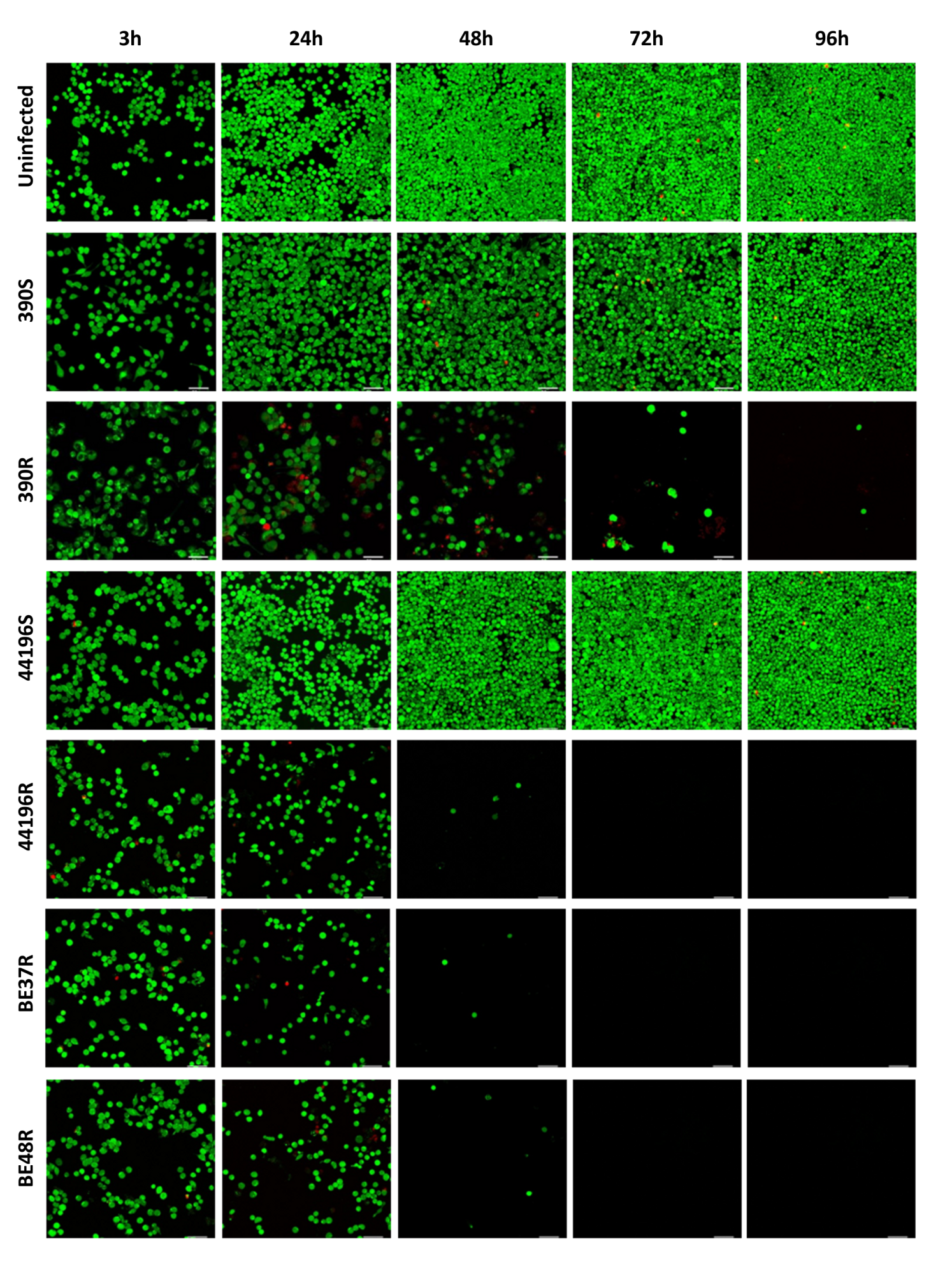


**Supplementary Fig. 1:** Representative images of macrophages infected with R and S morphotypes of M. abscessus, and uninfected macrophages, at different time points after infection. Macrophages were labelled with green fluorescent Calcein acetoxymethyl (viable cells) and red fluorescent Ethidium homodimer-1(unviable cells). Bar size 50 µm.
